# Development of a Novel Classification for Gastric Antral Vascular Ectasia Based on Upper Gastrointestinal Endoscopy Findings in Patients With Gastrointestinal Bleeding and Anemia

**DOI:** 10.7759/cureus.79472

**Published:** 2025-02-22

**Authors:** Ajay Patwa, Virendra Atam, Ajay K Mishra, Abhishek Singh, Priya Mishra, Archana Devi, Anurag Chaudhary, Isha Atam, Gunjan Arora, Pragya LNU

**Affiliations:** 1 Medicine, Gastroenterology and Hepatology Unit, King George’s Medical University, Lucknow, IND; 2 Internal Medicine, King George’s Medical University, Lucknow, IND; 3 Hepatology, Sanjay Gandhi Postgraduate Institute of Medical Sciences, Lucknow, IND; 4 Community Medicine and Public Health, King George’s Medical University, Lucknow, IND; 5 Medicine, King George’s Medical University, Lucknow, IND; 6 Physiology, King George’s Medical University, Lucknow, IND

**Keywords:** anemia, artificial intelligence, classification, gastrointestinal bleeding, grade, upper gastrointestinal endoscopy

## Abstract

Background and aim: Upper GI endoscopy (UGIE) identifies diverse findings in GI bleeding (GIB) patients. Gastric antral vascular ectasia (GAVE), a notable GIB cause, lacks a comprehensive classification. Besides, a detailed photographic data bank is crucial for training novice endoscopists and developing artificial intelligence (AI)-driven neural networks. This study aims to compile a databank of UGIE findings in GIB patients and propose a novel GAVE classification.

Materials and methods: A retrospective analysis of prospectively collected data was manually performed. Case records were evaluated, including video and photographic UGIE findings and indications for GIB patients. Fully and partially completed records were included; grossly incomplete ones were excluded. Data on common findings, indications, laboratory parameters, and risk factors were analyzed and classified. A novel classification of GAVE was developed.

Results: Of 1821 records, 430 were analyzed. The mean patient age was 44.5 ± 16.8 years (range 10-85), with males comprising 251 (58.4%). The primary indication for UGIE was anemia (388, 90.2%), followed by hematemesis (57, 13.3%). The most frequent endoscopic findings were varices (139, 32.3%) and portal hypertensive gastropathy (131, 30.5%). GAVE was present in 22 (5.1%). Key risk factors included non-vegetarian diet (102, 23.7%) and alcohol consumption (101, 23.5%).

Conclusions: This study highlights the most common indications, findings, and risk factors for UGIE in GIB patients, such as anemia, varices, and non-vegetarian diet, respectively. It provides a novel classification of GAVE and establishes a resourceful photographic data bank for beginner training and AI applications, enhancing diagnostic and therapeutic approaches in GIB.

## Introduction

GI bleeding (GIB) and anemia are common but serious medical conditions that often require prompt investigation to prevent significant morbidity and mortality. Upper GI bleeding (UGIB), which originates from the esophagus, stomach, or duodenum, is one of the leading causes of hospital admissions, especially in emergency settings. The presentation can range from overt bleeding, such as hematemesis and melena, to chronic occult bleeding that results in iron deficiency anemia. While GIB can result from various etiologies, ranging from peptic ulcer disease to esophageal varices, its diagnosis often necessitates the use of upper GI endoscopy (UGIE), which remains the gold standard for identifying the source of the bleeding [1-3].

Gastric antral vascular ectasia (GAVE) is the dilatation of vascular channels at the gastric antrum. It is a significant cause of GIB and anemia. Its various endoscopic phenotypes have been reported [4-6]. However, it lacks a comprehensive classification.

Anemia, frequently associated with GIB, can result from both acute hemorrhage and chronic blood loss. In patients presenting with anemia without overt GI symptoms, UGIE often uncovers a range of underlying causes, including erosive gastritis, peptic ulcers, and malignancies, many of which might have gone undiagnosed otherwise [3]. The ability of endoscopy to directly visualize the mucosa allows clinicians to identify and manage these lesions early, which is crucial for improving outcomes in these patients.

In the Indian subcontinent, UGIB and anemia are frequently encountered in clinical practice, with a wide spectrum of underlying etiologies. The prevalence and causes of UGIB can vary depending on local healthcare access, endemic conditions such as *Helicobacter pylori* infection, and socioeconomic factors [4-8]. However, limited studies have focused on systematically documenting the spectrum of UGIE findings in patients presenting with both GIB and anemia in this region, making it crucial to understand their endoscopic profile.

Besides, a beginner in GI endoscopy or artificial intelligence (AI)-based neural network requires a large pool of data sets and validated grades and classifications of findings for training to identify images and plan therapeutic intervention [9-12].

This study aims to evaluate the range of UGIE findings in patients presenting with GIB and anemia at a tertiary care center in North India. The objectives were to analyze indications, laboratory parameters, common findings classified by standard grades, and common endoscopic interventions to manage them. By doing so, we hope to provide insights into the prevalent causes of GIB and guide strategies for the diagnosis, prevention, and management of these conditions in the regional population, as well as to provide a large pool of data sets for beginners in GI endoscopy or an AI-based neural network for training. AI in medicine is integrated into enhancing diagnosis, personalizing treatment, accelerating drug discovery, improving patient monitoring, and optimizing healthcare workflows, leading to better outcomes. Our study will reduce costs and increase accessibility to quality care.

## Materials and methods

Study design and setting

This study was a retrospective analysis of prospectively collected data conducted at the endoscopy laboratory of the Department of Medicine, King George Medical University, Lucknow, India. The study included record sheets of patients who underwent endoscopy between June 14, 2021, and July 10, 2024. All datasheets of patients undergoing UGIE for various indications were included in the analysis. The study was approved by the Institutional Ethical Committee of King George's Medical University (approval number: 2501/Ethics/2024).

Inclusion and exclusion criteria

**Figure 1:**
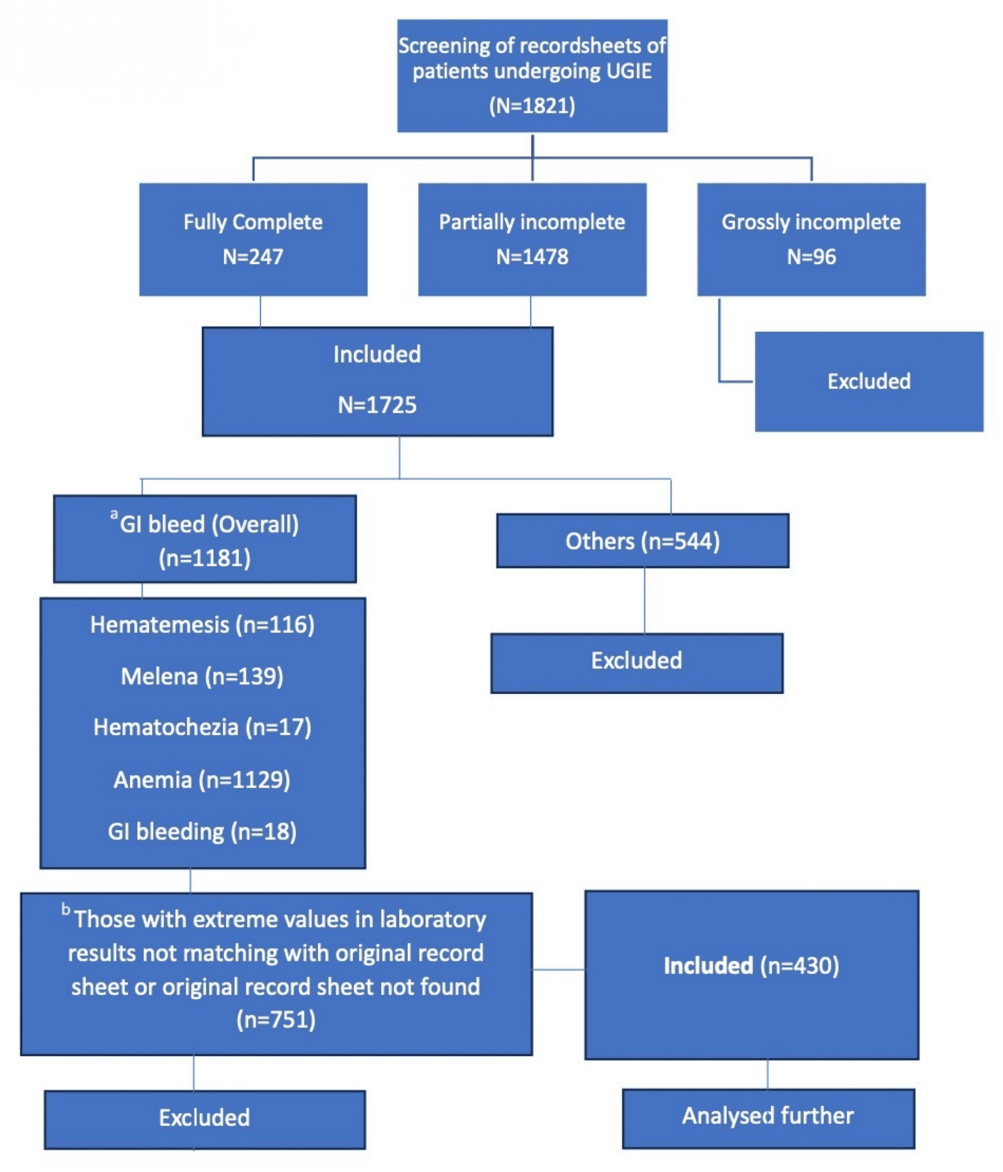
Study flowchart ^a ^GIB is a subheading and an indication, as it was mentioned as an indication in a few cases. ^b ^Total number exceeds the number of patients, as many patients had more than one indication. GIB: gastrointestinal bleeding, UGIE: upper gastrointestinal endoscopy, GI: gastrointestinal

All datasheets in which either GIB (upper or lower) or symptoms of GIB (hematemesis, melena, hematochezia, or anemia) were written as indications were included. Anemia was defined as an indication of UGIE if written as such or any other complaint with hemoglobin less than 12 gm/dl [13]. Only fully complete or partially incomplete datasheets were analyzed further. A complete datasheet was defined as all the columns of study variables in the Excel sheet (Microsoft Corporation, Redmond, WA, USA) being fully filled up, and partially incomplete data was defined as at least both UGIE findings and indications being filled up (Figure 1).

All other record sheets of patients presenting with symptoms and indications other than above (i.e., hematemesis, melena, hematochezia, and anemia), e.g., abdominal pain, chest pain, generalized weakness, dysphagia, etc., and those with grossly incomplete data, were excluded. Grossly incomplete data was defined as any column among the two main study variables, i.e., UGIE findings or indications were not filled up in the Excel sheet. The data of patients with extreme values in laboratory results that did not match the original record sheet or whose original record sheet was not found were excluded (Figure 1).

Standard operating procedure for managing patients with GIB and anemia

Managing GIB and anemia at our institute follows a structured approach for early diagnosis, stabilization, and treatment. Patients undergo an initial evaluation, including a detailed history, physical examination, and laboratory tests like complete blood count, iron studies, and liver function tests. Immediate stabilization involves securing IV access, fluid resuscitation, and blood transfusion if needed, especially for patients with hemoglobin levels below 7 g/dL. Proton pump inhibitors are administered in suspected peptic ulcer disease, while vasoactive agents and antibiotic prophylaxis are used in suspected variceal bleeding.

UGIE is performed within 24 hours in unstable or actively bleeding patients. In setups where UGIE is unavailable, patients should be promptly referred to a higher facility for UGIE as early as possible after stabilization. High-risk, past-bleeder, or actively bleeding esophageal varices or non-variceal lesions (e.g., ulcers) undergo therapeutic interventions per standard guidelines [14]. These interventions include endoscopic variceal ligation (EVL) or endoscopic sclerotherapy (EST) for esophageal varices, glue injection for gastric varices, or hemostasis techniques (e.g., adrenaline injection, thermal coagulation, electrical coagulation, or argon plasma coagulation) for non-variceal bleeding. Post-procedural monitoring involves serial hemoglobin checks and close observation for rebleeding.

Management also focuses on addressing underlying conditions such as chronic liver disease, and patients receive lifestyle counseling on alcohol, tobacco, and dietary modifications.

Data collection and outcomes

Endoscopy was performed by one experienced endoscopist and one trainee endoscopist. Indications/symptoms for UGIE, common findings, baseline laboratory parameters, therapeutic procedures, and their outcomes were recorded on a predesigned pro forma and entered in an Excel sheet. Endoscopic videos and photographs were recorded using Scopydoc software (Medsynaptic Pvt Ltd, Pune, India). Standard terms and definitions were used to define endoscopic lesions. Standard grades and classifications were used for varices, ulcers, reflux esophagitis, hiatus hernia, and corrosive injury [15-23]. Mild gastritis was defined as mild mucosal erythema. In contrast, severe gastritis was defined as severe mucosal erythema, erosions, ecchymosis, or frank bleeding after slight modification from the original grading described elsewhere [24].

Statistical analysis and data presentation

Data quality was checked by three authors and classified as fully complete, partially incomplete, and grossly incomplete, as mentioned above. Shapiro-Wilk test was applied to test the normal distribution of data. Since a few data points (serum creatinine, bilirubin, aspartate aminotransferase (AST), and alanine aminotransferase (ALT)) were not normally distributed, all data were presented in tables as frequency (if categorical) and mean, median, mode, standard deviation (SD), and range as continuous.

## Results

Indications, risk factors, and laboratory parameters

The mean age of patients was 44.5 ± 16.8 years (range: 10-85); 251 (58.4%) were males and 179 (41.6%) were females. The most common indication in patients undergoing endoscopy for GIB was anemia (90.2%), followed by hematemesis (13.3%) and melena (13%) (Table 1).

**Table 1:** Common indications/symptoms in patients undergoing endoscopy (N=430) The overall percentage is more than 100 because several patients had more than one indication. GIB: gastrointestinal bleeding

SN	Indications	n (%)
1	Anemia	388 (90.2)
2	Hematemesis	57 (13.3)
3	Melena	56 (13.0)
4	GIB	7 (1.6)
5	Hematochezia	6 (1.4)

The most common risk factor associated with GIB was a non-vegetarian diet (23.7%), followed by alcohol consumption (23.5%) (Table 2).

**Table 2:** Risk factors (N=430) HTN: hypertension, DM: diabetes mellitus, TB: tuberculosis

SN	Risk factor	n (%)
1	Non-vegetarian food	102 (23.7)
2	Alcohol	101 (23.5)
3	Tobacco	92 (21.4)
4	Smoking	55 (12.8)
5	DM	51 (11.9)
6	Past surgeries	49 (11.4)
7	HTN	34 (7.9)
8	TB	10 (2.3)
9	Allergy	3 (0.7)

Laboratory parameters that showed abnormal range were hemoglobin (8.78 ± 2.15), total bilirubin (2.58 ± 5.12 mg/dl), AST (75.27 ± 155.38 IU/ml), ALT (59.24 ± 177.34 IU/ml), and serum albumin (3.27 ± 1.26). The rest of the parameters were within the normal range (Table 3). Two record sheets with anemia had positive occult blood in the stool.

**Table 3:** Laboratory parameters at presentation (N=430) Hb: hemoglobin, TLC: total leukocyte count, AST: aspartate aminotransferases, ALT: alanine transaminases, ALP: alkaline phosphatase, PT: prothrombin time, INR: international normalized ratio

SN	Parameter	Unit	Mean	Median	Mode	SD	Range
1	Hb (n=384)	g/dL	8.78	8.90	10.4	2.15	3.5-16.3
2	TLC (n=382)	cells/µL	7,691.24	6,690.0	6,100	3,871.48	400-17,760
3	Platelet count (n=382)	lakhs/µL (or 10^9/L)	1.99	1.73	1	0.95	0.8-4.99
4	Serum creatinine (n=311)	mg/dL	1.46	0.94	0.8	1.54	0.24-14.9
5	Bilirubin (total) (n=322)	mg/dL	2.58	0.82	0.46	5.12	0.14-35.7
6	AST (n=314)	IU/L	75.27	37	26	155.38	4.2-1655
7	ALT (n=298)	IU/L	59.24	27.25	22	177.34	1.43-2271
8	ALP (n=303)	IU/L	272.36	229.1	121	180.88	17-1289
9	Serum protein (n=236)	g/dL	6.28	6.20	6	1.54	2.23-18
10	Serum albumin (n=238)	g/dL	3.27	3.14	3	1.26	1-15
11	PT (n=184)	Seconds	16.48	16	16	2.63	11.6-22.6
12	INR (n=168)	Ratio	1.25	1.21	1	0.26	0.52-2.05

Endoscopic findings

The most common endoscopic findings in patients undergoing UGIE were varices (32.3%), followed by portal hypertensive gastropathy (PHG) (30.5%) (Table 4). Common miscellaneous findings (56, 13%) were esophageal stricture (5, 1.2%), corrosive injury (11, 2.6%), esophagitis (9, 2.1%), hiatus hernia (4, 0.9%), and esophageal candidiasis (3, 0.7%).

**Table 4:** Common findings in UGIE in patients of GIB (N=430) The overall percentage is more than 100 because several patients had more than one finding. PHG: portal hypertensive gastropathy, GAVE: gastric antral vascular ectasia, GERD: gastroesophageal reflux disease, UGIE: upper gastrointestinal endoscopy, GIB: gastrointestinal bleeding

SN	Common findings	n (overall %)	Intra-group %
1	Varices	139 (32.3)	-
	Esophageal	123 (28.6)	88.5
	Gastric	16 (3.7)	11.5
2	Ulcers	17 (3.9)	-
	Esophageal	3 (0.7)	17.6
	Gastric	10 (2.3)	58.8
	Duodenal	4 (0.9)	23.5
3	PHG	131 (30.5)	-
4	Gastritis	70 (16.3)	-
5	Polyp	4 (0.9)	-
6	Abnormal growth	13 (3.0)	-
	Esophageal	3 (0.7)	23.1
	Gastric	6 (1.4)	46.2
	Gastroesophageal	1 (0.2)	7.7
	Duodenal	3 (0.7)	23.1
7	GAVE	22 (5.1)	-
8	Miscellaneous	56 (13.0)	-
9	Normal	105 (24.4)	-

Out of the 123 patients with esophageal varices, 35 (28.5%) presented with red color signs (RCS), while 88 (71.5%) had no RCS. Grade I varices were highest in frequency (59, 48%). Among 16 patients with gastric varices, only one (6.3%) had RCS, while 15 (93.7%) did not. Gastroesophageal varices type 2 (GOV2) were found in the largest frequency (6, 37.5%) among them (Table 5).

**Table 5:** Detailed description of varices (N=430) F1-3: fundal varices type 1, 2, and 3; GOV1 and GOV2: gastroesophageal varices type 1 and type 2; IGV1 and IGV2: isolated gastric varices type 1 and type 2; RCS: red color sign

SN	Type	Overall	With RCS	Without RCS
Esophageal varices		123 (100)	35 (28.5)	88 (71.5)
1	I	59 (48.0)	2 (3.4)	57 (96.6)
2	II	26 (21.1)	6 (23.1)	20 (76.9)
3	III	38 (30.9)	27 (71.1)	11 (28.9)
Gastric varices		16 (100)	1 (6.3)	15 (93.7)
1	GOV1	5 (31.3)	1 (20)	4 (80)
	F1	2 (12.5)	1 (50)	1 (50)
	F2	3 (18.8)	0	3 (100)
	F3	0	0	0
2	GOV2	6 (37.5)	0	6 (100)
	F1	2 (12.5)	0	2 (100)
	F2	4 (25)	0	4 (100)
	F3	0	0	0
3	IGV1	4 (25)	0	4 (100)
	F1	1 (6.3)	0	1 (100)
	F2	2 (12.5)	0	2 (100)
	F3	1 (6.3)	0	1 (100)
4	IGV2	1 (6.3)	0	1 (100)

Of the 17 patients with ulcers, the majority were gastric ulcers (10, 58.82%), followed by duodenal ulcers (4, 23.52%) and esophageal ulcers (3, 17.64%). Grade III ulcers were found in the largest frequency (10, 58.8%) (Table 6).

**Table 6:** Details of ulcers (Forrest grading)

SN	Type	Esophageal (3, 17.6%)	Gastric (10, 58.8%)	Duodenal (4, 23.5%)	Overall (N= 17, 100 %)
1.	Ia	1	0	0	1
2.	Ib	0	2	1	3
3.	IIa	0	1	0	1
4.	IIb	0	0	0	0
5.	IIc	0	0	1	1
6.	III	2	7	2	11

Of the 11 patients with corrosive injury, the majority were esophageal injuries (6, 54.5%), followed by gastric injuries (4, 36.4%) and duodenal injuries (1, 9.1%) (Table 7).

**Table 7:** Details of corrosive injury (Zargar grading)

SN	Type	Esophageal (6, 54.5%)	Gastric (4, 36.4%)	Duodenal (1, 9.1%)	Overall (N=11, 100 %)
1.	I	1	0	1	2
2.	IIA	4	2	0	6
3.	IIB	0	1	0	1
4.	IIIA	1	1	0	2
5.	IIIB	0	0	0	0
6.	IV	0	0	0	0

The representative photographs of the endoscopic findings are given in Figures 2-18. Among patients who had abnormal growth, esophageal growth was seen in three (5.36%), gastric growth in six (10.72%) patients, duodenal in three (5.36%) patients, and gastroesophageal growth in one (1.78%) patient.

**Figure 2:**
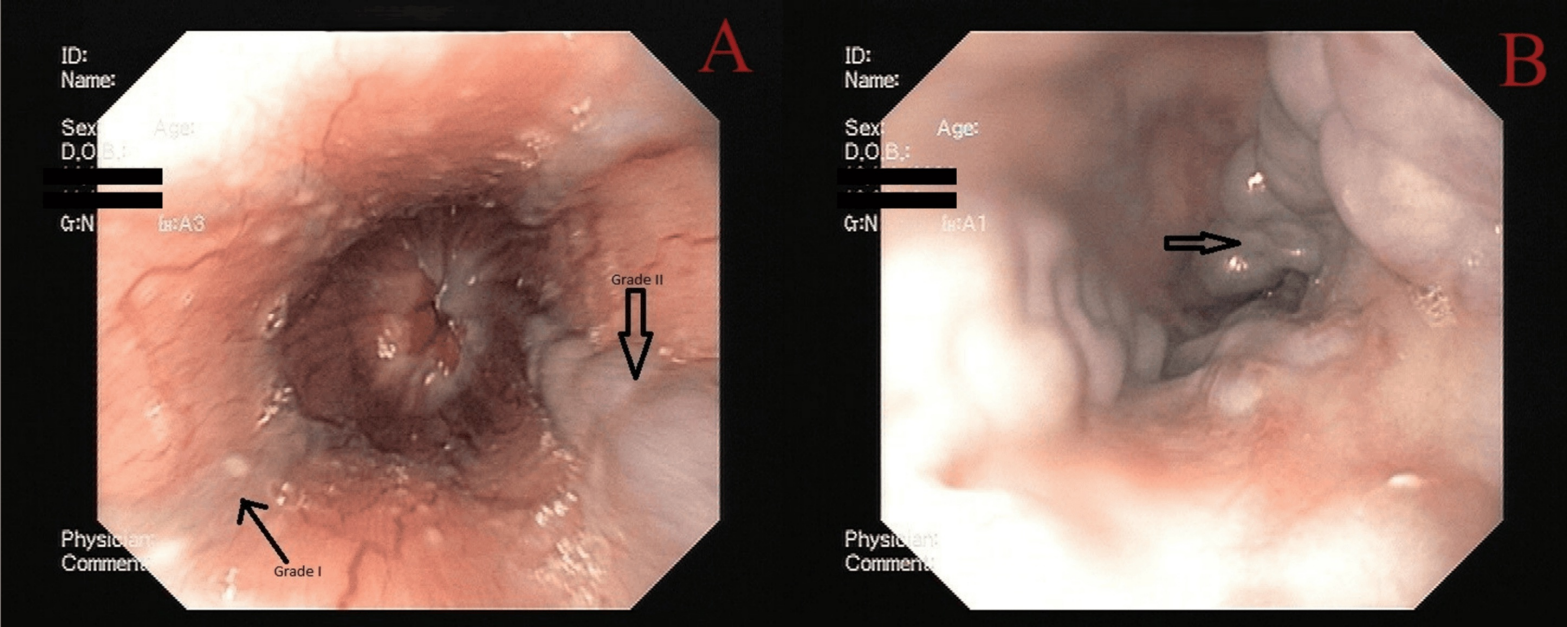
Esophageal varices: (A) Grade I (narrow arrow), Grade II (broad arrow), and (B) Grade III (broad arrow) Esophageal varices (modified Paquet classification) (A) Grade I: varices extending just above the mucosal level, and Grade II: varices projecting by one-third of the luminal diameter that cannot be compressed with air insufflation. (B) Grade III: varices projecting up to 50% of the luminal diameter or in contact with each other.

**Figure 3:**
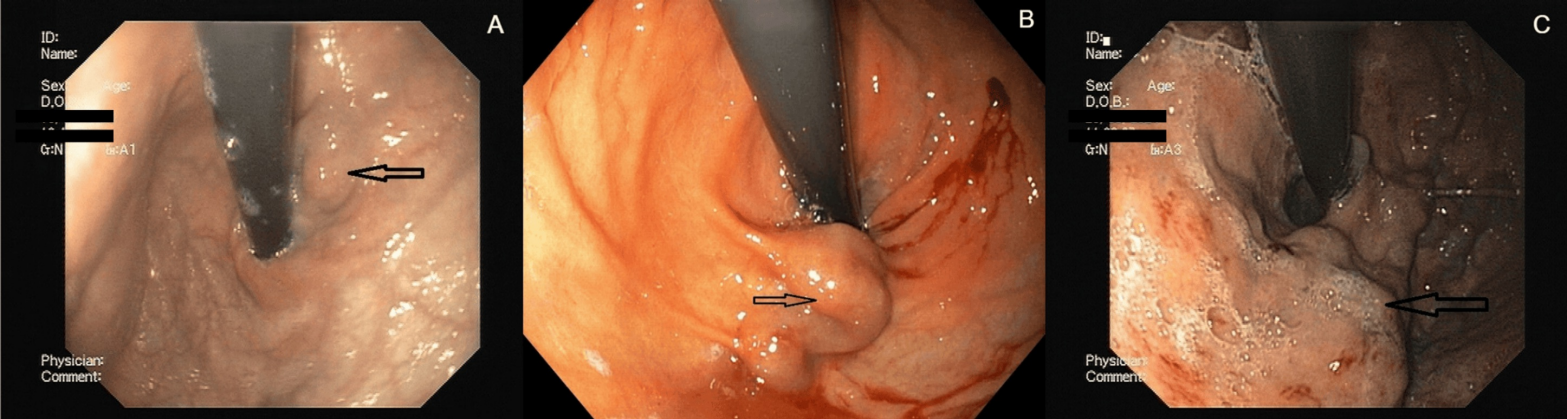
GOV21: (A) GOV1F1, (B) GOV1F2, and (C) GOV1F3 GOV1 (combined sarin and hoshizome classification) (A) GOV1F1: varices extend toward the lesser curvature of the stomach, with a tortuous appearance. (B) GOV1F2: varices extend toward the lesser curvature of the stomach, with a nodular appearance. (C) GOV1F3: varices extend toward the lesser curvature of the stomach, with a tumorous appearance. GOV1: gastroesophageal varices type 1

**Figure 4:**
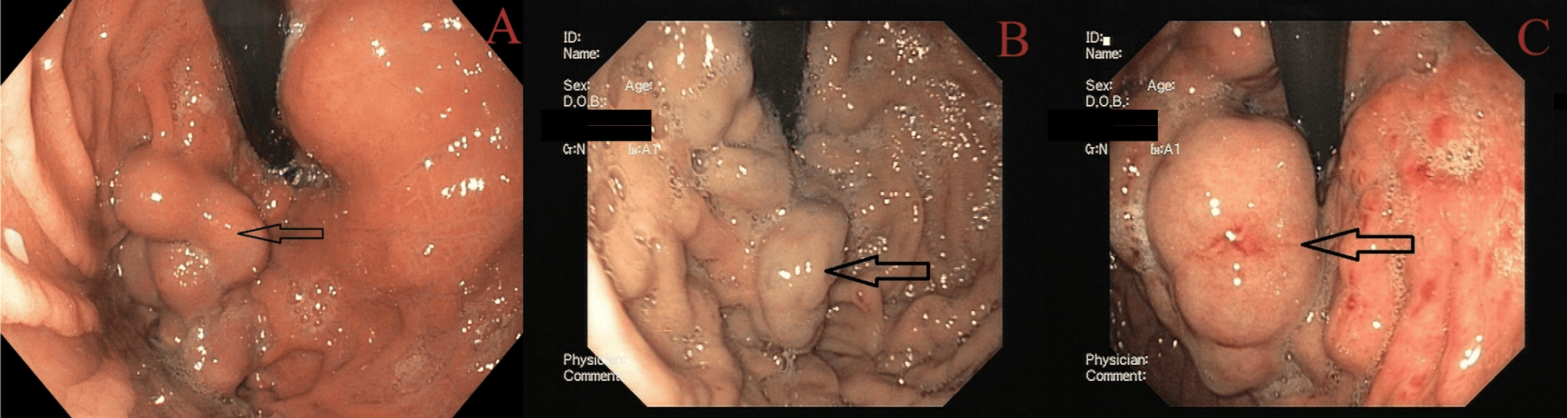
GOV2: (A) GOV2F1, (B) GOV2F2, and (C) GOV2F3) GOV2 (combined sarin and hoshizome classification) (A) GOV2F1: varices extend toward the gastric fundus with a tortuous appearance. (B) GOV2F2: varices extend toward the gastric fundus with a nodular appearance. (C) GOV2F3: varices extend toward the gastric fundus with a tumorous appearance. GOV2: gastroesophageal varices type 2

**Figure 5:**
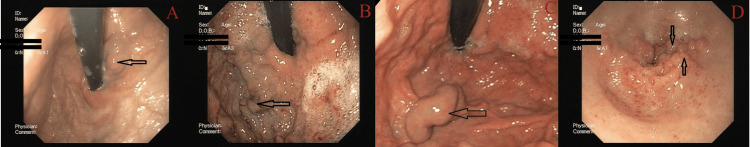
Isolated gastric varices: (A) IGV1F1, (B) IGV1F2, (C) IGV1F3, and (D) IGV2 Isolated gastric varices (combined sarin and hoshizome classification) (A) IGV1F1: varices in the fundus with a tortuous appearance. (B) IGV1F2: varices in the fundus with a nodular appearance. (C) IGV1F3: varices in the fundus with a tumorous appearance. (D) IGV2: varices elsewhere in the stomach, like the body, antrum, or pylorus.

**Figure 6:**
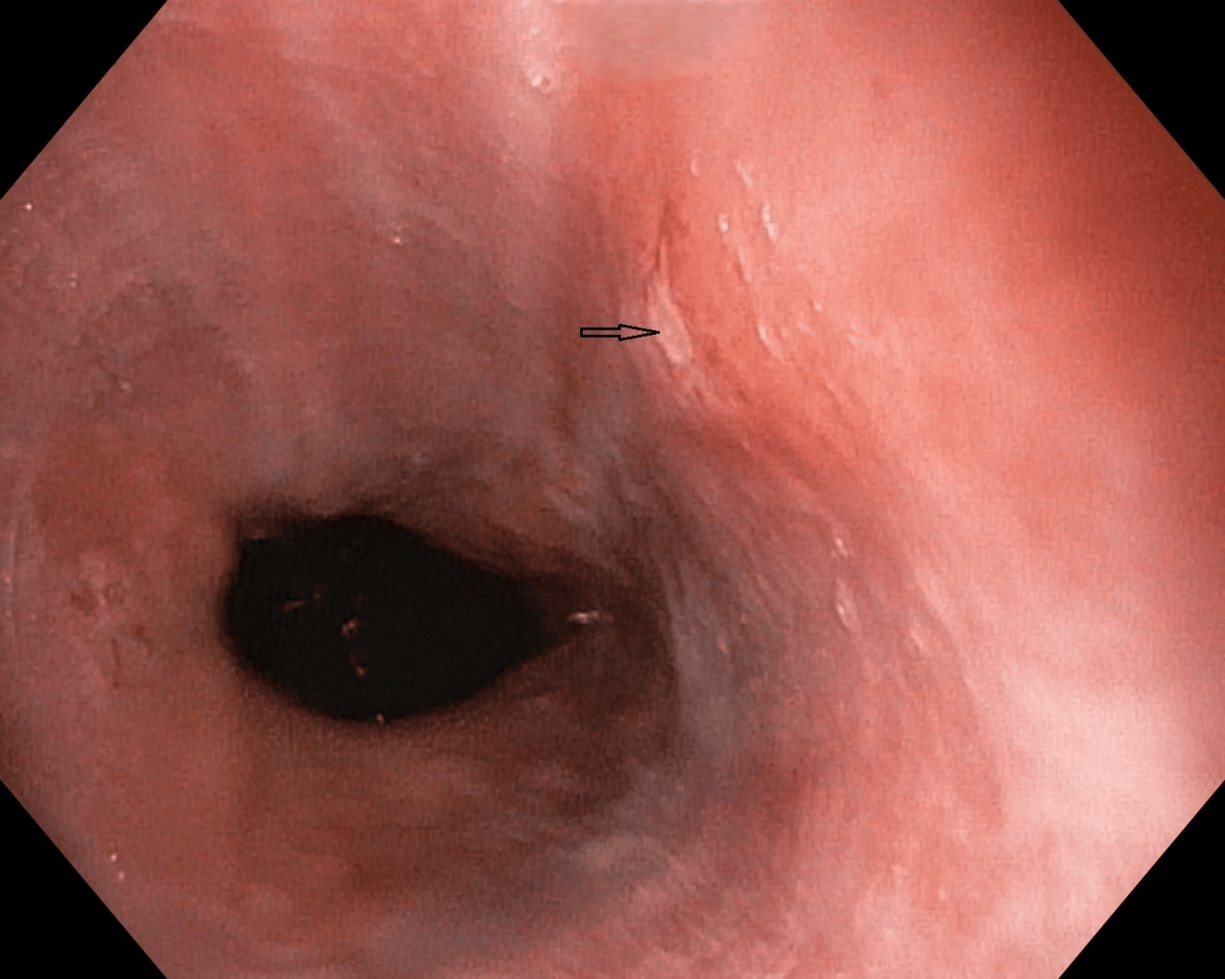
Esophageal ulcer

**Figure 7:**
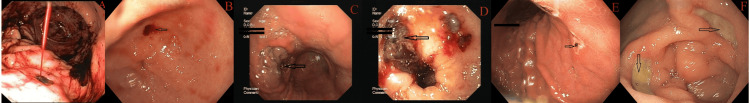
Gastric ulcer: (A) Grade Ia, (B) Grade 1b, (C) Grade IIa, (D) Grade IIb, (E) Grade IIc, and (F) Grade III Gastric ulcer (Forrest classification) (A) Grade Ia: active spurting. (B) Grade 1b: active oozing. (C) Grade IIa: non-bleeding visible vessels. (D) Grade IIb: adherent clot. (E) Grade IIc: flat spot in the ulcer base. (F) Grade III: clean base ulcer.

**Figure 8:**
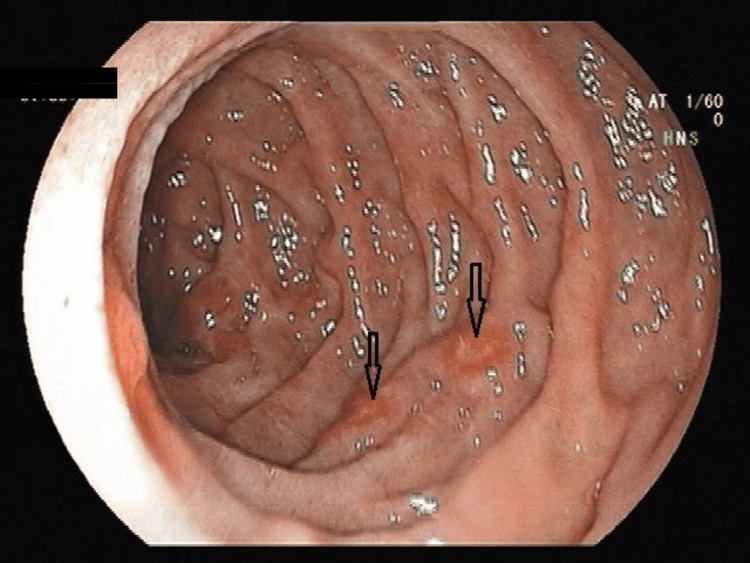
Duodenal ulcer

**Figure 9:**
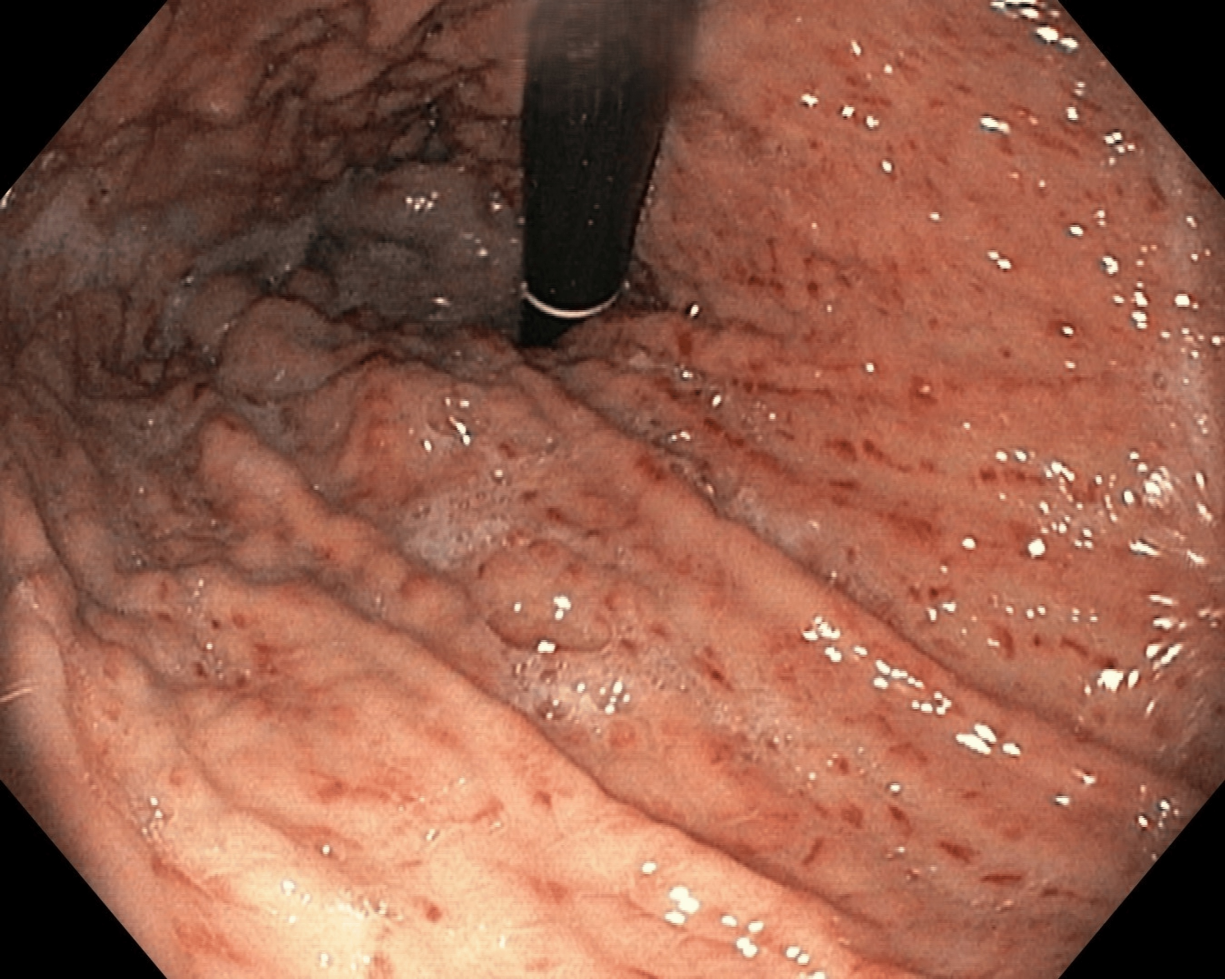
PHG PHG: portal hypertensive gastropathy

**Figure 10:**
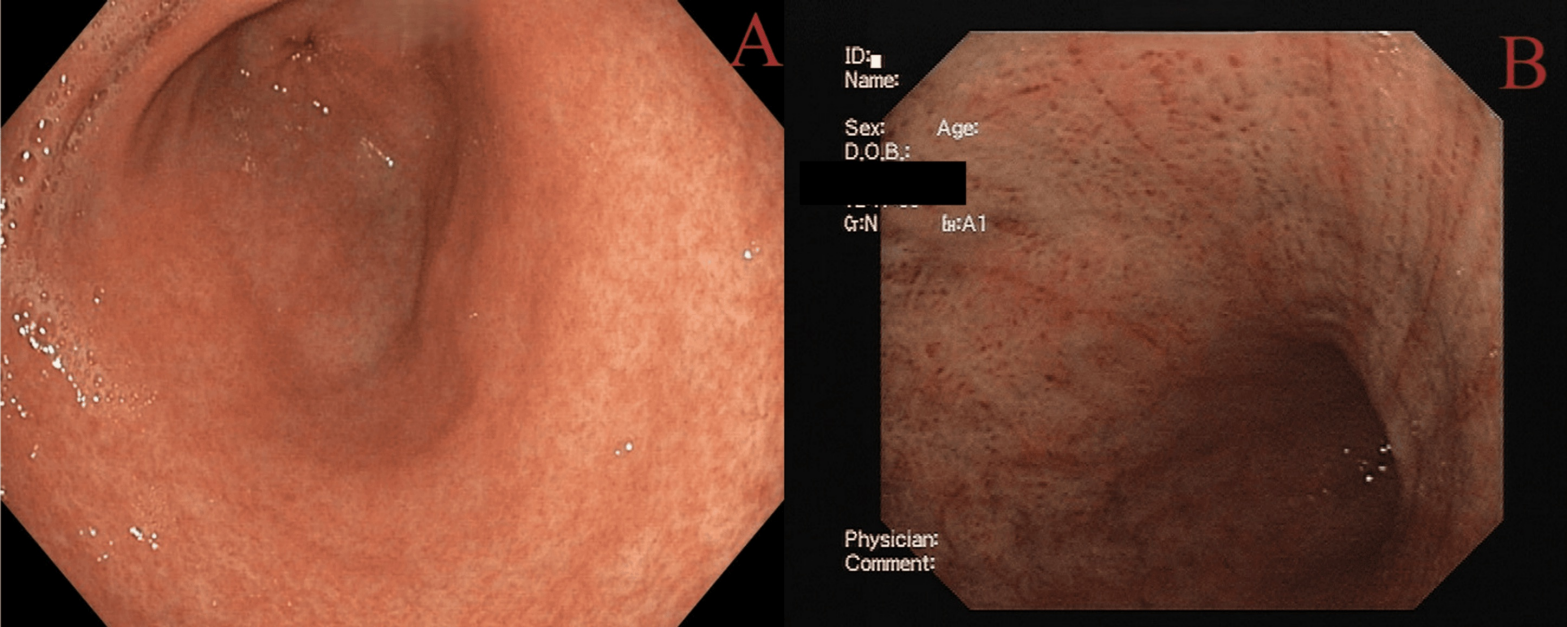
Gastritis: (A) mild gastritis and (B) severe gastritis

**Figure 11:**
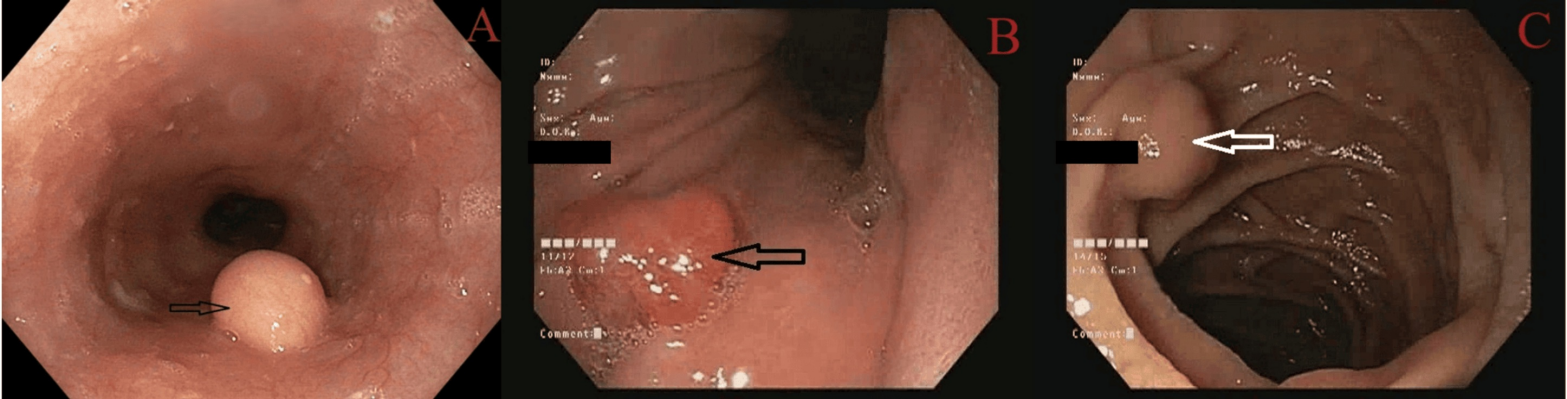
Polyps: (A) esophageal, (B) gastric, and (C) duodenal polyp

**Figure 12:**
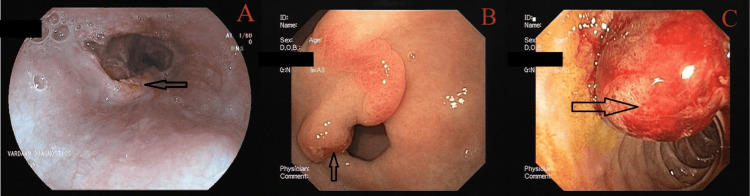
Abnormal growths: (A) esophageal, (B) gastric, and (C) duodenal

**Figure 13:**
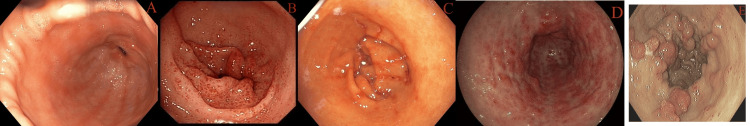
GAVE: (A) linear, (B) punctate, (C) hypertrophic, (D) ulcerated, and (E) nodular GAVE: gastric antral vascular ectasia

**Figure 14:**
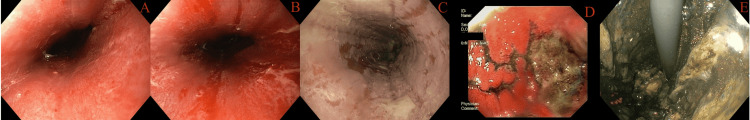
Corrosive injury: (A) Grade I, (B) Grade IIA, (C) Grade IIB, (D) Grade IIIA, and (E) Grade IIIB Corrosive injury (Zargar classification) (A) Grade I: edema and erythema. (B) Grade II A: hemorrhage, erosions, blisters, and superficial ulcers with exudate. (C) Grade II B: deep focal or circumferential ulcers. (D) Grade III A: focal necrosis with multiple deep ulcers with brown, black, or gray discoloration. (E) Grade III B: extensive necrosis.

**Figure 15:**
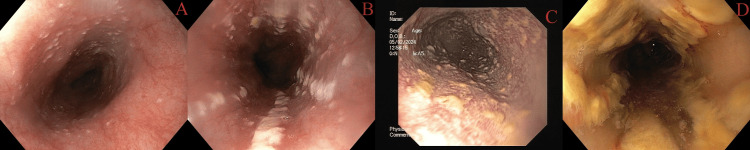
Esophageal candidiasis: (A) Grade I, (B) Grade II, (C) Grade III, and (D) Grade IV Esophageal candidiasis (Kodsi classification) (A) Grade I: plaques <2 mm, no edema and ulcerations. (B) Grade II: plaques >2 mm, edema, possible no ulcerations. (C) Grade III: linear and confluent plaques and no ulcerations. (D) Grade IV: Grade III plus detachment of mucous membranes and luminal narrowing.

**Figure 16:**
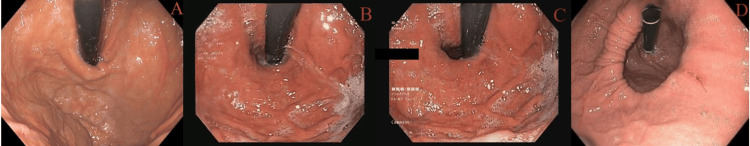
Hiatus hernia: (A) Hill’s Grade I, (B) Hill’s Grade II, (C) Hill’s Grade III, and (D) Hill’s Grade IV Hiatus hernia (Hill’s classification) (A) Hill’s Grade I: wall-like gastroesophageal flap valve, always with tight closure around the endoscope. (B) Hill’s Grade II: gastroesophageal flap valve hardly present anymore, no closure around the endoscope. (C) Hill’s Grade III: gastroesophageal flap valve less marked, with respiration-dependent incomplete closure around the endoscope. (D) Hill’s Grade IV: gastroesophageal flap valve no longer present, permanent opening of the esophagogastric junction.

**Figure 17:**
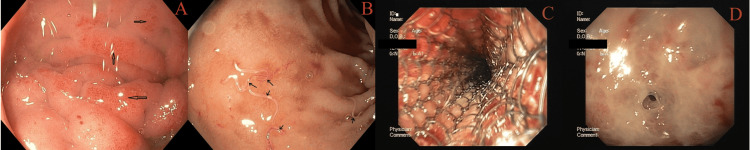
Miscellaneous: (A) duodenopathy, (B) hook worm in duodenum, (C) esophageal stent, and (D) pyloric stenosis

**Figure 18:**
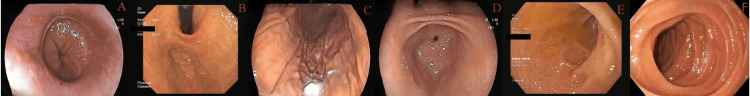
Normal mucosa: (A) esophagus, (B) gastric fundus, (C) gastric body, (D) gastric antrum and pylorus, (E) first part of the duodenum, and (F) second part of the duodenum

Development of a novel, comprehensive classification of GAVE (King George's Medical University classification of GAVE)

We observed at least five distinct phenotypes of GAVE (n=22, 5.1%) in our study: linear (14, 63.6%), punctate (2, 9.0%), ulcerated (2, 9.0%), hypertrophic (1, 4.5%), and nodular (3, 13.6%) (Figure 13). Based on this observation, we proposed a novel, comprehensive classification of GAVE (King George's Medical University classification of GAVE): (1) linear, (2) punctate, (3) ulcerated, (4) hypertrophic, and (5) nodular.

Therapeutic interventions and outcome

EVL was done in 162 of all 1,821 patients (8.8%) with esophageal varices. Cyanoacrylate glue was injected in 11 out of all 1821 patients (0.6%) with gastric varices. Five esophageal varices and one gastric varix were actively bleeding during the endoscopy. The bleeding was successfully controlled after EVL or glue injection. Only one patient with a duodenal ulcer had active bleeding, in which an adrenaline injection followed by argon plasma coagulation was done with a successful outcome. One of the four polyps underwent polypectomy by snare, while the rest of the three polyps were biopsied using punch biopsy forceps. All growths were biopsied by punch biopsy forceps, and biopsy specimens were sent for histopathological examination. Two patients with gastric varices had severe bleeding after cyanoacrylate injection that stopped spontaneously after two and three minutes. No other major complication was seen during any diagnostic or therapeutic procedure.

## Discussion

This study presents a comprehensive overview of the UGIE findings in patients with GIB and anemia conducted at a tertiary care center in North India. The analysis of 430 record sheets revealed significant insights into these patients' prevalence, risk factors, and clinical characteristics. These findings hold importance for clinical practice and the training of beginner endoscopists, as well as the development of AI-based diagnostic tools.

Common endoscopic findings

The most common finding was varices (32.5%), followed by PHG (30.5%). The high prevalence of varices and PHG reflects the common association of chronic liver disease, particularly cirrhosis, with upper GIB in the region. This pattern is in line with other studies where liver disease remains a leading cause of GI bleeding due to portal hypertension [7,9,10,25]. Furthermore, the finding of non-variceal causes such as ulcers (3.9%) and gastritis (16.3%) underscores the multifactorial etiology of GIB [2,26]. The presence of esophageal and gastric varices highlights the need for skilled endoscopists to perform interventions like EVL and cyanoacrylate glue injection for gastric varices, both of which were employed in the study. Our study found five distinct phenotypes of GAVE. Based on this observation, we proposed a novel, comprehensive classification of GAVE. Other studies have given only a partial classification of GAVE or a description of case studies [4-6].

Clinical indications for endoscopy

Anemia was the most common indication for endoscopy (90.2%), followed by overt bleeding symptoms such as hematemesis (13.3%) and melena (13.0%). This distribution suggests that many patients present with chronic or occult bleeding, manifesting as anemia rather than acute, overt symptoms. These patients had almost a variety of lesions ranging from simple erosions to frank malignant growth, as mentioned in the existing literature [27,28]. The high incidence of anemia indicates the need for timely intervention in patients with unexplained hemoglobin reduction. Early diagnosis and treatment could potentially prevent more severe complications.

GAVE is an important cause of UGIB and anemia. The novel classification of GAVE may provide beginner endoscopists with awareness of the various phenotypes. It can also provide a useful base for further studies on the correlation of severity of anemia in different types of GAVE.

Risk factors

Among the recorded risk factors, consuming a non-vegetarian diet (23.7%) and alcohol (23.5%) were the most significant. These findings support existing literature where dietary habits, especially high intake of red meat and alcohol consumption, are strongly associated with the development of varices, gastritis, and other GI pathologies [29]. Tobacco use and smoking, which are also risk factors for various GI conditions [30], were present in about 21.4% and 12.8% of the cases, respectively. This aligns with broader public health data suggesting a link between lifestyle factors and GI diseases.

Laboratory parameters

The laboratory findings further illuminate the clinical condition of these patients. Hemoglobin levels averaged 8.78 g/dL, confirming this cohort's significant burden of anemia. Abnormal liver function tests, as demonstrated by elevated AST and ALT levels, reinforce the role of liver disease in GIB. Elevated bilirubin levels (2.58 ± 5.12 mg/dL) point toward hepatic dysfunction, potentially from cirrhosis or other chronic liver conditions. This underlines the importance of comprehensive patient assessment, including liver function, in cases of GIB.

Clinical implications

The study’s findings have important implications for clinical practice. First, objective grading and classification of endoscopic findings are important for beginners and experienced endoscopists. This study provides all standard grades and classifications in one place with practical examples. Second, the high prevalence of varices and PHG suggests that liver disease screening and management should be a priority in patients presenting with GIB, especially in resource-limited settings where cirrhosis and hepatitis are endemic. Third, the predominance of anemia as an indication for endoscopy highlights the need for clinicians to adopt a high index of suspicion for GIB in patients with unexplained anemia.

The data also suggest that lifestyle interventions targeting diet, alcohol, and tobacco use could play a role in reducing the incidence of GIB. The observed risk factor profile can guide public health initiatives aimed at prevention. Moreover, the recorded endoscopic findings provide a valuable dataset for beginners' training in GI endoscopy, allowing them to recognize common conditions and plan appropriate interventions.

Study limitations

While this study provides valuable data, there are several limitations to consider. Being a retrospective analysis, it relies on the accuracy and completeness of the recorded data. Although the study included fully and partially complete records, some cases of grossly incomplete data were excluded, which might have affected the overall representation. Furthermore, the exclusion of patients with non-GIB indications could have overlooked certain coexisting conditions that may influence the bleeding risk. Another limitation is that the study was conducted at a single institution, which may limit the generalizability of the findings to other regions with different epidemiological profiles.

Future directions

Given the findings of this study, future research could focus on prospective studies to validate these results further. Given the growing interest in AI-based diagnostics, there is also an opportunity to explore the use of AI intelligence in the automated identification of endoscopic images of varices, PHG, and other common findings. GAVE is a significant cause of GIB and anemia. Further studies may be planned to correlate the severity of anemia with various phenotypes of GAVE. Expanding the dataset to include multi-center data could enhance the findings' applicability and help develop more generalized guidelines for managing GIB.

## Conclusions

Our study establishes a valuable dataset of common endoscopic findings and indications, serving as a resource for training beginners in GI endoscopy and enhancing AI applications in this field. It also gives a novel and comprehensive classification of GAVE and valuable insight into risk factors for GIB and anemia.
